# Effect of long-term serum sodium levels on the prognosis of patients on maintenance hemodialysis

**DOI:** 10.1080/0886022X.2024.2314629

**Published:** 2024-02-18

**Authors:** Siyu Chen, Bin Pan, Xiaowei Lou, Jianghua Chen, Ping Zhang

**Affiliations:** a Kidney Disease Center, the First Affiliated Hospital, College of Medicine, Zhejiang University, Hangzhou, Zhejiang Province, China; b Key Laboratory of Kidney Disease Prevention and Control Technology, Hangzhou, Zhejiang Province, China; c National Key Clinical Department of Kidney Disease, Hangzhou, Zhejiang Province, China; d Institute of Nephrology, Zhejiang University, Hangzhou, Zhejiang Province, China; e Zhejiang Clinical Research Center of Kidney and Urinary System Disease, Hangzhou, Zhejiang Province, China

**Keywords:** Low sodium, high sodium, hemodialysis, risk factors, prognosis

## Abstract

Abnormal serum Na (SNa) levels are common in patients with chronic kidney disease (CKD) which is associated with increased morbidity and mortality. There are relatively few studies on the effect of SNa indicators on the prognosis of patients undergoing maintenance hemodialysis (MHD). We aim to investigate the effect of long-term SNa levels on the survival and prognosis of patients undergoing hemodialysis (HD). Newly entered HD patients in the registration system of Zhejiang Provincial Dialysis Quality Control Center between January 1, 2010 and December 31, 2019 were included and followed up until December 31, 2020. Multiple sodium levels were collected from patients, defining long-term SNa as the mean of multiple SNa, according to which patients were grouped, with the prognostic differences between subgroups compared by Kaplan-Meier modeling and multifactorial Cox regression modeling. Finally, a total of 21,701 patients were included in this study and Cox regression showed that decreased SNa levels (Na < 135 mmol/L, HR = 1.704, 95% CI 1.408–2.063, *p* < 0.001; 135≦Na≦137.5 mmol/L, HR = 1.127,95% CI 1.016–1.250, *p* = 0.024) and elevated SNa levels (142.5 < Na≦145mmol/L, HR = 1.198, 95% CI 1.063–1.350, *p* = 0.003; Na > 145mmol/L, HR = 2.150, 95% CI 1.615–2.863, *p* < 0.001) were all independent risk factors for all-cause mortality in MHD patients.

## Introduction

SNa plays an important role in maintaining the volume of extracellular fluid, regulating acid-base balance, and maintaining normal osmolality of plasma, and also participates in the process of excitatory transmission of nerve and muscle junctions. Previous studies have shown that about 30%-40% of hospitalized patients will suffer from disturbed serum sodium concentration, which produces a series of undesirable clinical consequences, including unsteady gait, rhabdomyolysis, bone fracture, and infections, resulting in a significant increase in the mortality rate of patients [1–5].

Disturbed SNa concentrations are correlated with mortality in patients with CKD at different stages of the disease. Studies have shown that in non-dialysis-dependent patients with CKD, low or high sodium at baseline is associated with higher mortality rates and is independent of other conditions known to cause hyponatremia [6–8]. In contrast, relatively few studies have been conducted on MHD patients as affected by SNa [9,10]. Some prospective studies on HD patients have suggested that reduced SNa levels in patients are associated with increased all-cause and cardiovascular mortality [11–13]. Rhee et al. reported a U-shaped relationship between SNa and all-cause mortality in HD patients, with SNa <138 mmol/L or ≧144 mmol/L being associated with a higher risk of death, even after adjusting for relevant factors such as inter-dialysis weight gain, blood urea nitrogen and glucose levels, the relationship remained [14]. A retrospective study including 178,114 MHD patients further counted the metric of pre- and post-dialysis SNa change in a single dialysis session and found for the first time that higher pre- and post-dialysis SNa change values were associated with a higher mortality rate in patients undergoing HD [15]. However, no study has further investigated the relationship between long-term sodium levels during HD and patient prognosis.

In this study, we retrospectively analyzed the indicators of newly admitted patients undergoing MHD, with the aim of exploring the impact of long-term SNa levels on the survival prognosis of HD patients.

## Materials and methods

### Study participants

The study participants were newly enrolled HD patients from January 1, 2010 to December 31, 2019 in the registration system of Zhejiang Dialysis Quality Control Center. The inclusion criterias were: (1) age ≧18 years; (2) patients receiving MHD with dialysis duration ≧3 months. The exclusion criteria were: (1) patients with information missing or obviously wrong, including gender, age, time of first dialysis, vascular access; (2) patients with previous peritoneal dialysis or hemodialysis combined with peritoneal dialysis; (3) history of previous renal transplantation; and (4) patients with temporary hemodialysis due to acute renal injury and other reasons. The time of the patient’s first dialysis was taken as the starting point of follow-up, and the end point of follow-up was until December 31, 2020, with the follow-up period including those who died, converted to peritoneal dialysis or kidney transplantation.

### Baseline covariates

General data of patients were collected, including age, gender, primary disease (including chronic glomerulonephritis, diabetic nephropathy, hypertensive nephropathy, polycystic kidney, obstructed kidney), comorbidities (including diabetes mellitus, cardiovascular disease, cerebrovascular disease, and tumors), modality of vascular access (including autologous arteriovenous fistula, temporary intubation, long-term intubation, and graft vascular fistula). The combined cardiovascular diseases were defined as patients diagnosed with coronary atherosclerotic heart disease, myocardial infarction, heart failure, arrhythmia, cardiomyopathy, and others; the combined cerebrovascular diseases were defined as patients diagnosed with stroke including cerebral hemorrhage, cerebral infarction, cerebral embolism, and others; and the combined tumors were defined as patients whose primary disease was not a tumor, but accompanied with a tumor disease.

Average clinical data from the first three months after the patient’s first dialysis were used as baseline data, with only values prior to each dialysis session being recorded, including: sodium (Na), calcium (Ca), phosphorus (P), potassium (K), fasting glucose (Glu), serum creatinine (Scr), blood urea nitrogen (BUN), uric acid (UA), albumin (Alb), glutamic pyruvic transaminase (ALT), glutamic oxaloacetic transaminase (AST), total bilirubin (TB), alkaline phosphatase (Alp), parathyroid hormone (PTH), white blood cell count (WBC), hemoglobin (Hb), and platelet count (Plt). In this regard, the average Na level of first three months after the start of dialysis was defined as early SNa.

The mean Na level before a single dialysis session was calculated every 3 months thereafter, starting at the time of the patient’s first dialysis session. Patients with less than 3 recordings were excluded, with the final average Na level over the entire dialysis period defined as the long-term SNa level. All patients were divided into the following 6 groups as determined by long-term SNa levels: group 1 (Na < 135mmol/L), group 2 (135≦Na≦137.5 mmol/L), group 3 (137.5 < Na≦140mmol/L), group 4 (140 < Na≦142.5 mmol/L), group 5 (142.5 < Na≦145mmol/L), and group 6 (Na > 145 mmol/L). The following indices were measured: the coefficient of ­variation of long-term SNa (CV), defined as the $\frac{\text{standard deviation of long}-\text{term SNa}}{\text{mean of long}-\text{term SNa}};$ the rate of SNa variation (RV), defined as $\frac{\left|\text{mean long}-\text{term SNa value}-\text{early SNa value}\right|}{\text{early SNa value}}\times 100\text{\%}.$The quantity of SNa variation (QV), comparing the magnitude of multiple fluctuations in SNa at 140 mmol/L after the patient started on HD, was the mean of the $\left|\text{pre}-\text{dialysis SNa values}-140.\right|$

### Statistical analysis

Normality test and variance chi-square test were used for the measurement data, which were expressed as mean ± standard deviation for normal distribution and t-test for comparison between the groups, while median (quartile, three-quarter quartile) for non-normally distributed measurements and Kruskal-Wallis test for comparison between the groups. The counting data were expressed as the number of cases (percentage) and χ^2^ used for comparison between the groups. Comparison of survival rates and survival curves between groups with different SNa levels was performed by Kaplan–Meier model and log-rank test. Multifactorial Cox regression was used to analyze the correlation with mortality between long-term SNa levels and their indicators of change, with some variables included further categorized as follows: age (≧60 years; <60 years), primary disease (non-chronic glomerulonephritis; chronic glomerulonephritis), vascular access (non-autologous arteriovenous fistula; autologous arteriovenous fistula). RCS (Restricted Cubic Spline, RCS) was used to fit the relationship between SNa level with risk of death. *p* < 0.05 was considered statistically different. All analyses were statistically processed using SPSS 23.0 software (IBM®) and R version 4.2.2.

## Results

**Figure 1.**
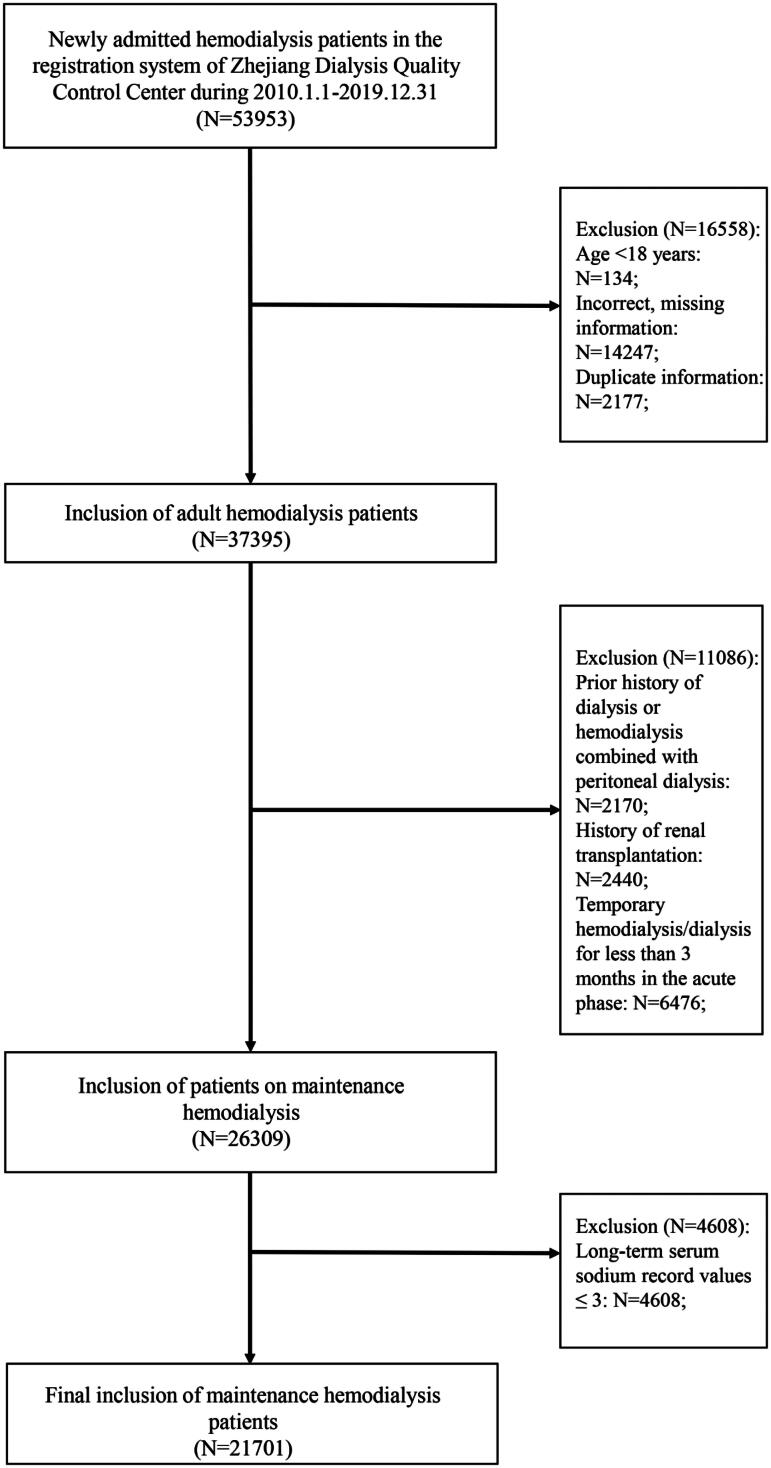
Patient selection flow chart.

It was a retrospective cohort study. A total of 53,953 newly entered HD patients in the registration system of Zhejiang Dialysis Quality Control Center from January 1, 2010 to December 31, 2019 were enrolled in this study. According to the inclusion criteria, 134 patients aged <18 years, 2177 duplicate registered patients, 14247 patients with missing or incorrect basic information, and 2170 patients with a history of previous peritoneal dialysis or hemodialysis combined with peritoneal dialysis, 2440 patients with a history of renal transplantation, and 6476 patients who underwent temporary hemodialysis in the acute phase were excluded, resulting in the final enrollment of 26309 patients. Excluding 4608 patients with long-term SNa record values of less than 3 times, 21701 patients were finally included, as shown in Figure 1.

Among 21,701 patients, a total of 11,738 (54%) patients developed hyponatremia (Na < 135 mmol/L) based on early SNa levels, of which 3679 (17%) patients had Na ≤ 130 mmol/L, while 1,362 (5%) patients developed hypernatremia (Na > 145 mmol/L). A total of 394 (2%) patients developed hyponatremia and 196 (1%) patients developed hypernatremia depending on the mean of long-term SNa level. Six groups were further divided as shown in Table 1. There were statistically significant differences between the groups in terms of gender, age, primary renal disease, combined diabetes mellitus, and cerebrovascular disease, whereas no statistical differences were found in terms of combined cardiovascular disease, combined tumor, and vascular access. A higher proportion of patients in the Na < 135 mmol/L group had comorbid diabetes mellitus, whereas the age of patients with Na > 145 mmol/L was significantly greater than that of the other groups.

**Table 1.** Baseline information on maintenance hemodialysis patients grouped according to long-term serum sodium levels (*N* = 21701).

Characteristics	Na < 135 (*N* = 394)	135≦Na≦137.5 (*N* = 2577)	137.5 < Na≦140 (*N* = 8374)	140 < Na≦142.5 (*N* = 8110)	142.5 < Na≦145 (*N* = 2050)	Na > 145 (*N* = 196)	*P*-value
Male (%)	233(59)	1497(58)	5008(60)	4291(61)	1288(63)	114(58)	0.028
Age (years)	60.50 ± 13.85	60.54 ± 13.91	58.54 ± 15.03	58.65 ± 15.22	60.35 ± 15.11	64.07 ± 14.35	<0.001
Primary disease (%)							<0.001
chronic glomerulonephritis	133(34)	1120(44)	4341(52)	4607(57)	1120(55)	77(39)	
diabetic nephropathy	214(54.3)	1043(40.5)	2292(27.4)	1533(18.9)	344(16.8)	36(18.4)	
hypertensive nephropathy	1(0.3)	83(3.2)	396(4.7)	462(5.7)	126(6.1)	14(7.1)	
polycystic kidney	4(1.0)	65(2.5)	332(4.0)	468(5.8)	146(7.1)	7(3.6)	
obstructed kidney	6(1.5)	33(1.3)	98(1.2)	116(1.4)	32(1.6)	6(3.1)	
others	36(9.1)	233(9.0)	915(10.9)	924(11.4)	282(13.8)	56(28.6)	
Comorbidities (%)							
diabetes mellitus	250(64)	1198(47)	2682(32)	1866(23)	394(19)	43(22)	<0.001
cardiovascular disease	169(43)	1037(40)	3336(40)	3205(40)	804(39)	88(45)	0.484
cerebrovascular disease	25(6)	195(8)	541(7)	510(6)	149(7)	21(11)	0.032
tumors	15(4)	115(5)	427(5)	376(5)	108(5)	13(7)	0.325
Modality of vascular access (%)							0.178
autologous arteriovenous fistula	133(34)	862(33)	2907(35)	2840(35)	660(32)	56(29)	
temporary intubation	180(45.7)	1096(42.5)	3564(42.6)	3410(42.0)	874(42.6)	83(42.3)	
long-term intubation	79(20.1)	609(23.6)	1847(22.1)	1815(22.4)	504(24.6)	54(27.6)	
graft vascular fistula	2(0.5)	8(0.3)	46(0.5)	37(0.5)	9(0.4)	3(1.5)	
others	0(0.0)	2(0.1)	10(0.1)	8(0.1)	3(0.1)	0(0.0)	
Laboratory Values							
Na (mmol/L)	126.74 ± 8.88	131.07 ± 6.57	133.91 ± 6.13	136.19 ± 6.16	138.44 ± 6.95	141.25 ± 9.56	<0.001
Ca (mmol/L)	2.17(2.00,2.30)	2.14(2.00,2.30)	2.14(1.99,2.29)	2.15(1.99,2.30)	2.14(2.01,2.29)	2.14(2.01,2.31)	0.642
P (mmol/L)	1.57(1.19,1.96)	1.53(1.22,1.95)	1.56(1.21,1.97)	1.56(1.22,1.98)	1.55(1.20,1.96)	1.62(1.27,1.96)	0.418
K (mmol/L)	4.56 ± 0.87	4.56 ± 0.90	4.57 ± 0.89	4.56 ± 0.90	4.58 ± 0.90	4.51 ± 0.90	0.879
Glu (mmol/L)	8.58 ± 4.49	7.39 ± 3.44	6.58 ± 2.55	6.17 ± 2.04	6.07 ± 2.00	6.09 ± 1.84	<0.001
Scr (μmol/L)	579.67 ± 258.52	633.81 ± 265.81	671.86 ± 272.96	678.58 ± 273.79	659.25 ± 264.11	590.79 ± 255.13	<0.001
BUN (mmol/L)	20.11 ± 9.44	20.45 ± 8.21	21.10 ± 8.27	21.09 ± 8.00	21.09 ± 7.98	19.84 ± 6.71	0.007
UA (μmol/L)	385.1 ± 116.64	392.32 ± 112.71	398.38 ± 112.99	400.09 ± 121.48	399.84 ± 105.52	396.6 ± 109.48	0.099
Alb (g/L)	32.94 ± 5.45	33.93 ± 5.13	34.69 ± 5.28	35.32 ± 5.26	35.11 ± 5.44	34.72 ± 6.41	<0.001
ALT (U/L)	15.00(11.00,22.00)	13.67(10.00,20.88)	14.49(10.00,21.33)	14.50(10.00,21.67)	14.50(10.44,21.27)	15.46(11.15,22.00)	<0.001
AST (U/L)	20.00(15.03,25.75)	18.00(14.00,24.00)	18.27(14.17,24.25)	18.46(14.25,24.00)	18.33(14.33,24.00)	19.55(14.68,25.84)	<0.001
TB (μmol/L)	5.68(4.15,7.51)	5.70(4.33,7.70)	5.80(4.38,7.80)	5.78(4.30,7.80)	5.70(4.23,7.63)	6.33(4.50,8.40)	0.523
Alp (U/L)	85.00(69.00,118.00)	83.10(66.00,108.37)	81.50(64.35,106.00)	80.00(63.59,104.06)	81.50(65.00,104.00)	85.51(65.05,108.94)	0.042
PTH (pg/ml)	151.31(77.65,258.18)	181.90(96.50,300.37)	200.40(108.91,331.80)	207.55(109.44,352.93)	224.32(122.49,377.90)	195.00(109.25,297.83)	<0.001
WBC (x10^9^/L)	6.70 ± 1.96	6.86 ± 2.68	6.63 ± 2.74	6.62 ± 4.36	6.49 ± 2.15	6.84 ± 2.49	0.023
Hb (g/L)	86.85 ± 15.36	89.00 ± 16.05	87.90 ± 15.20	87.85 ± 14.80	87.00 ± 14.55	87.08 ± 15.82	0.001
Plt (x10^9^/L)	188.36 ± 69.69	189.93 ± 67.05	179.86 ± 63.62	176.4 ± 66.23	168.05 ± 63.12	161.6 ± 53.49	<0.001

**Figure 2.**
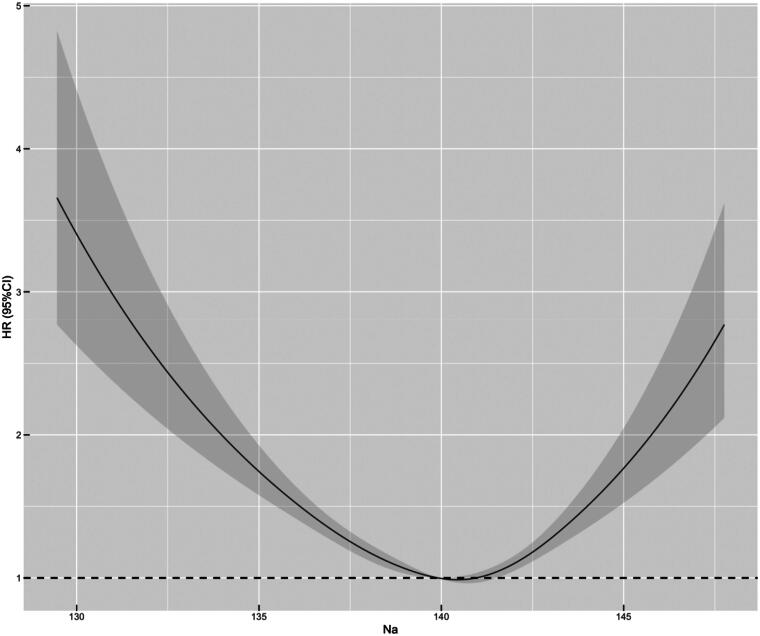
Curve relationship between mean long-term SNa level with HR for all-cause mortality. SNa level is used as a continuous variable on the x-axis, and black curve represents the median adjusted HR, while gray portion represents the 95% confidence interval of the adjusted HR. Na: serum sodium level.

**Figure 3.**
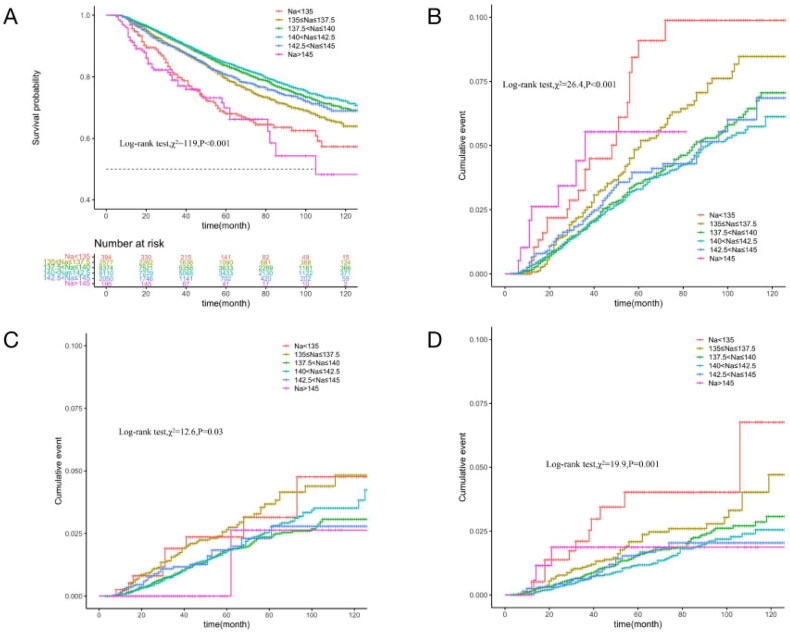
Comparison of survival by cause of death in groups with different long-term SNa levels in MHD patients (Kaplan–Meier survival curves). The horizontal coordinate is the follow-up time (months) and the vertical coordinate is the cumulative survival rate or mortality rate. A: All-cause mortality; B: Cardiovascular deaths; C: Infection deaths; D: Cerebrovascular death.

Average long-term SNa levels were used as a continuous variable to fit the relationship between SNa levels and mortality risk with an RCS curve. As shown in Figure 2, the HR of all-cause mortality increased with either lower or higher SNa levels, with optimum SNa levels near 140 mmol/L. The relationship between long-term SNa levels and survival was analyzed by Kaplan-Meier survival curves, which showed that the cumulative survival rate for all-cause mortality (Log-rank test, χ2 = 119, *p* < 0.001) was significantly lower in SNa levels < 135 mmol/L and >145 mmol/L than in the other groups. Cumulative mortality from cardiovascular, cerebrovascular, and infectious deaths was further compared between the groups. The curves showed that cardiovascular mortality (Log-rank test, χ2 = 26.4, *p* < 0.001) was significantly higher in patients with SNa levels ≤137.5 mmol/L and >145 mmol/L than in the other groups, while cerebrovascular mortality (Log-rank test, χ2 = 19.9, *p* = 0.001) and infection mortality (Log-rank test, χ2 = 12.6, *p* = 0.03) were significantly lower in patients with SNa levels ≤137.5 mmol/L, as shown in Figure 3.

The effect of long-term SNa level on prognosis was analyzed by multifactorial Cox regression modeling with 140 < Na≦ 142.5 mmol/L as the reference, which showed that without adjusting each parameter, decreased SNa levels (Na < 135 mmol/L, HR = 2.056, 95% CI 1.702-2.483, *p* < 0.001; 135≦Na≦137.5 mmol/L, HR = 1.334,95% CI 1.205–1.478, *p* < 0.001) and elevated SNa levels (142.5 < Na≦145mmol/L, HR = 1.249, 95% CI 1.108–1.407, *p* < 0.001; Na > 145mmol/L, HR = 2.610, 95% CI 1.96–-3.473, *p* < 0.001) were all independent risk factors for death in patients with MHD. After adjusting for sex, age, primary disease, comorbidities and vascular access, decreased (Na < 135 mmol/L, HR = 1.704, 95% CI 1.408–2.063, *p* < 0.001; 135≦Na≦137.5 mmol/L, HR = 1.127,95% CI 1.016–1.250, *p* = 0.024) and elevated SNa levels (142.5 < Na ≤ 145mmol/L, HR = 1.198, 95% CI 1.063–1.350, *p* = 0.003; Na > 145mmol/L, HR = 2.150, 95% CI 1.615–2.863, and *p* < 0.001) remained an independent risk factor for all-cause mortality in MHD patients, as shown in Table 2.

**Table 2.** Cox regression analysis of long-term serum sodium levels and all-cause mortality (*N* = 21701).

Na (mmol/L)	Model 1		Model 2	
	HR (95%CI)	*P-*value	HR (95%CI)	*P*-value
140 < Na ≤ 142.5	1		1	
Na < 135	2.056 (1.702–2.483)	<0.001	1.704 (1.408–2.063)	<0.001
135 ≤ Na ≤ 137.5	1.334 (1.205–1.478)	<0.001	1.127 (1.016–1.250)	0.024
137.5 < Na ≤ 140	1.070 (0.992–1.155)	0.082	1.013 (0.938–1.094)	0.743
142.5 < Na ≤ 145	1.249 (1.108–1.407)	<0.001	1.198 (1.063–1.350)	0.003
Na > 145	2.610 (1.961–3.473)	<0.001	2.150 (1.615–2.863)	<0.001

HR: hazard ratio; CI: confidence interval.

Model 1: unadjusted parameters;.

Model 2: Corrected variables included gender, age, primary disease, comorbidities, and vascular access.

Accounting for the effect of blood glucose, especially hyperglycemia, on serum sodium levels, we categorized patients according to blood glucose and further performed Cox subgroup analysis. After correcting for multifactorial factors, as shown in Supplementary Table 1, we observed that in the subgroup with fasting blood glucose >7, SNa < 135mmol/L and >142.5 mmol/L were independent risk factors for increased risk of death in patients with MHD, whereas in the subgroup with blood glucose≦7, the different levels of serum sodium did not have any significant correlation with the risk of death in patients with MHD.

**Figure 4.**
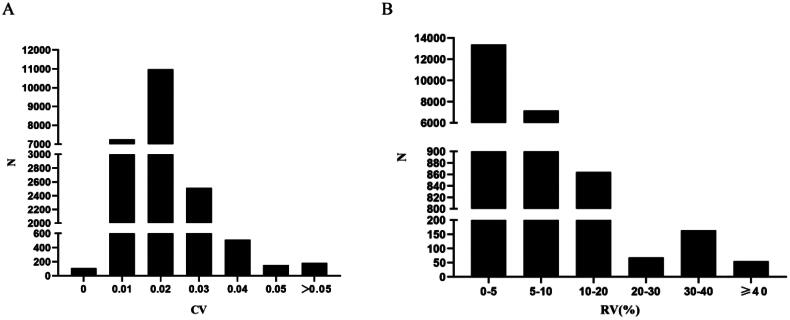
Distribution of the 21,701 patients in terms of the number of different indicators. N: numbers of cases. A: Coefficient of variation (CV), divided into the following six groups: CV = 0 (*N* = 107, 0.5%); CV = 0.01 (*N* = 7252, 33.4%); CV = 0.02 (*N* = 10981, 50.6%); CV = 0.03 (*N* = 2513, 11.6%); CV = 0.04 (*N* = 510, 2.4%); CV = 0.05 (*N* = 152, 0.7%); CV > 0.05 (*N* = 186, 0.8%); B: the rate of variation (RV) (%), divided into the following 6 groups: 0%≦RV < 5% (*N* = 13383, 61.7%); 5%≦RV < 10% (*N* = 7167, 33.0%); 10%≦RV < 20% (*N* = 864, 4.0%); 20%≦RV < 30% (*N* = 68, 0.3%); 30%≦RV < 40% (*N* = 164, 0.8%); 40%≦RV (*N* = 55, 0.2%).

Following indicators were further calculated: CV, which is the standard deviation/mean value of multiple SNa levels since the start of dialysis, found that the pre-dialysis SNa coefficient of variation in 21701 patients was 0.017 (0.014,0.022), with 84.5% of patients’ pre-dialysis SNa coefficients of variation ≤0.02, as shown in Figure 4. RV, as $\frac{\left|\text{mean long}-\text{term SNa value}-\text{early SNa value}\right|}{\text{early SNa value}}\times 100\text{\%},$ was found to be 4.3% (2.9%,6.0%) in 21,701 patients, while 94.7% of the patients had SNa variation <10%, as shown in Figure 4. QV, comparing the size of fluctuation of SNa level at 140 mmol/L for multiple times, found that the amount of SNa variation in 21,701 patients was 1.43 (0.66,2.45) mmol/L.

**Figure 5.**
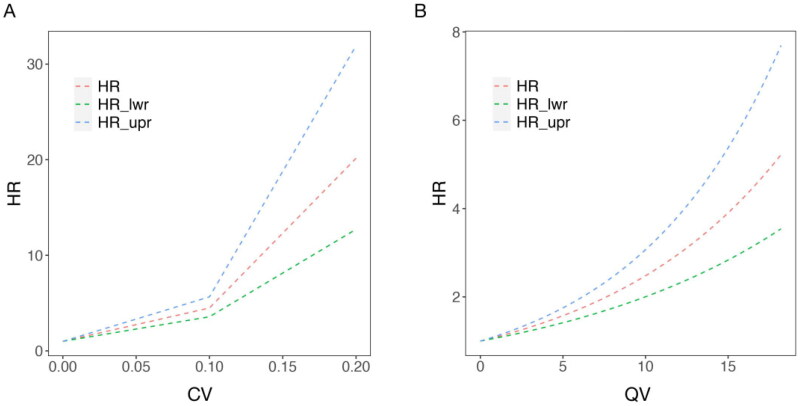
Curves of HR with each continuous variable in multifactorial Cox regression for long-term HD patients. In which, each index is used as a continuous variable on the X-axis, the red curve (HR) represents the adjusted HR, whereas the blue (HR_lwr) and green (HR_upr) curves represent the 95% confidence intervals of the HR. A: Curve of HR with the coefficient of variation (CV); B: Curve of HR with the quantity of SNa variation (QV).

The above three indicators were analyzed as continuous variables in multifactorial Cox regression analyses, respectively, incorporating gender, age, primary disease, and comorbidities. Results showed that after adjusting for the multivariate variables, CV and QV were associated with all-cause mortality HR, respectively, increasing with both (*p* < 0.001), as shown in Figure 5, whereas the rate of SNa variation did not affect the all-cause mortality rate of HD patients The COX results for all variables were shown in Table 3.

**Table 3.** Cox regression analysis of CV/QV and all-cause mortality (*N* = 21701).

Variables	Model 1		Model 2	
	HR (95%CI)	*P*-value	HR (95%CI)	*P*-value
CV(x10^5^)/QV	33.19 (3.325–331.2)	<0.001	1.095 (1.072–1.119)	<0.001
Gender (Female/Male)	0.972 (0.910–1.039)	0.408	0.976 (0.913–1.042)	0.462
Age (≧60/<60)	2.909 (2.701–3.133)	<0.001	2.954 (2.743–3.181)	<0.001
Primary disease (non-CKD/CKD)	1.414 (1.312–1.524)	<0.001	1.422 (1.319–1.533)	<0.001
Diabetes mellitus (Yes/No)	1.300 (1.205–1.402)	<0.001	1.297 (1.202–1.400)	<0.001
Cardiovascular disease (Yes/No)	1.118 (1.046–1.194)	<0.001	1.124 (1.052–1.200)	<0.001
Cerebrovascular diseas (Yes/No)	1.449 (1.302–1.612)	<0.001	1.455 (1.307–1.619)	<0.001
Tumors (Yes/No)	1.392 (1.226–1.589)	<0.001	1.386 (1.220–1.574)	<0.001

HR: hazard ratio; CI: confidence interval; CKD: chronic kidney disease;.

Model 1: Corrected variables included gender, age, primary disease, comorbidities (including diabetes mellitus, cardiovascular disease, cerebrovascular disease, and tumors) and CV;.

Model 2: Corrected variables included gender, age, primary disease, comorbidities (including diabetes mellitus, cardiovascular disease, cerebrovascular disease, and tumors) and QV.

## Discussion

Hyponatremia or hypernatremia is a common clinical condition and a complication of many diseases, the former of which is common in congestive heart failure, cirrhosis, and antidiuretic hormone secretion dysregulation syndrome, while the latter is more common in primary aldosteronism, different causes of cortisolism, and sodium retention due to traumatic brain injuries, cerebral vascular accidents, pituitary tumors, and other brain lesions. Abnormal SNa levels are more common in patients with CKD and patients with CKD are twice as likely to suffer from hyponatremia as non-CKD patients as data shows [16]. Considering the differences in definitions and populations, the prevalence of hyponatremia in hospitalized patients ranges from 2.5% to 30%, and in HD patients this percentage can reach up to 29.3% [5,11], whereas reports on the prevalence of hypernatremia in patients with CKD are relatively scarce. Our study retrospectively analyzed newly entered MHD patients in Zhejiang Province from 2010 to 2019, and ultimately found that 54% of patients had an early SNa level of <135 mmol/L (of which SNa <130 mmol/L accounted for 17%), and 6% >145 mmol/L. Whereas, during long-term follow-up during dialysis, 2% of patients had a mean pre-dialysis sodium level <135 mmol/L, and 0.9% >145 mmol/L. Patients were susceptible to hyponatremia at the early stage of dialysis, and the sodium level could be gradually corrected to the normal range in the course of long-term dialysis.

Previous studies have shown that both hyponatremia and hypernatremia are significantly associated with increased mortality in patients with different diseases [17–19]. Several studies have observed a correlation between hyponatremia and higher mortality in CKD patients undergoing HD [13,20,21]. Although high SNa has also been found to be associated with a higher risk of mortality in non-CKD patients [22,23], the correlation between hypernatremia and all-cause mortality has been less frequently addressed in studies of HD patients. In our study, we found that mortality in HD patients amounted to 18.8% during the follow-up period and that cardiovascular disease was the leading cause of death. After adjusting for age, primary morbidity, comorbidities, and other relevant indicators, long-term SNa levels showed a U-shaped relationship with patient prognosis, with either lower or higher SNa levels being an independent risk factor for all-cause mortality, and the greater the variability of the mean long-term SNa level fluctuating above and below 140 mmol/L, the worse the patient’s prognosis. Further, we calculated the rate of variation between long-term and early SNa levels and found that this indicator did not affect the prognosis of HD patients. Considering that patients’ low sodium levels in the early stage of dialysis can be gradually corrected with stable treatment of HD, we need to pay more attention to people with abnormal SNa indexes especially high sodium during long-term dialysis, to strengthen the monitoring and to make appropriate interventions when necessary.

Previous studies have reported that the risks of cerebral infarction, lower limb amputation, and hip fracture in HD patients are significantly increased with decreasing SNa levels [15]. A case-control study by Renneboog et al. found a high prevalence of falls due to mild chronic hyponatremia, which may be the result of gait and attention deficits [24]. SNa level is also a reliable and valid predictor of mortality in patients with enterocutaneous fistulae complicating sepsis [25]. Either too low or too high SNa levels increase the risk of infection, and hypernatremia is independently associated with mortality in patients with community-acquired pneumonia, especially in patients with hypertonic fluids for correction of water loss or inadequate rehydration [26], as well as in patients with hypernatremia, which significantly increases the mortality rate from bacterial infectious diseases [27]. Hypo- or hypernatremia, on the other hand, may also be a manifestation of some underlying disease associated with mortality. Patients could have inherent protein-energy depletion and inadequate solute intake that predispose them to excessive thirst, chronic cardiac insufficiency including combined ventricular hypertrophy, myocardial fibrosis, higher ultrafiltration volumes or rates during dialysis leading to intra-dialysis hypotension, myocardial shock and structural remodeling of the heart as well as diabetes mellitus, cerebral vascular accidents, cortisolism, and other related disorders. However, the mechanisms underlying the prognostic impact of serum sodium levels are not yet fully understood. Relevant studies suggest that increased plasma sodium concentration induces arterial structural remodeling, increased arterial oxidative stress, collagen deposition and luminal stenosis and further leads to wall thickening, which increases arterial tone and total peripheral vascular resistance, and ultimately leads to elevated blood pressure [28–30]. Extracellular hypertonicity also affects cellular function, thereby stimulating inflammatory responses in intestinal, bronchial and renal epithelial cells [31].

In our study, we further found that the coefficient of variation of long-term SNa levels affects the long-term prognosis of patients, and this indicator also reflects the level of sodium fluctuation in patients, with the higher coefficients of variation being associated with a worse prognosis. Some studies have shown a significant increase in the hospitalization rate of patients even when the change in serum sodium level is mild [32,33]. Related reports have shown that patients with spontaneous cerebral hemorrhage usually have electrolyte imbalance, and fluctuations in serum sodium concentration often indicate a poor prognosis [34]. A retrospective study in China this year similarly found that the occurrence of hypernatremia within 48 h of admission and large changes in serum sodium concentration within 48 h were risk factors for 28-day mortality in critically ill patients [35].

Studies have shown that weight gain during the interdialytic period is associated with increased mortality in patients, but the effect of serum sodium concentration has not been further considered [36,37]. We performed Cox subgroup analysis after grouping patients by glucose and found that the prognosis of the hyperglycemic population was more likely to be affected by sodium concentration. Ramdeen et al. also noted that the weight change of patients during the interdialysis period was mainly affected by sodium concentration and glucose, with patients who had excessive salt intake or high glucose were more likely to be thirsty to an increase in intake [38], which could lead to a high fluid load prior to the next dialysis session. Volume overload can lead to hemodynamic alterations in patients thereby affecting noncompliant cardiac structural variations, including left ventricular hypertrophy and fibrosis. The effect of serum sodium concentration on HD patients also needs to take the level of dialysate sodium concentration into account. Previous studies have shown that patients on hemodialysis may have an "osmolality or sodium setpoint," where patients with low or high pre-dialysis sodium concentrations will have a corresponding increase or decrease after each dialysis treatment [39,40]. In central nervous system, too rapid correction of serum sodium leads to osmotic demyelination syndrome, as brain cells have adapted to a hypotonic state during chronic hyponatremia, and once sodium supplementation is given at this point, it can lead to a prompt rise in plasma osmolality, resulting in dehydration of the brain tissue and secondary demyelination. There is also a significant association between excessive sudden sodium fluctuations before and after dialysis with stroke occurrence. Repeated sodium fluctuations and dialysis sessions resulted in cyclic osmolality variations, which may also be a factor affecting survival prognosis [41].

This study had some limitations. First, this was a retrospective analysis that could not clarify the causal relationship between disturbed sodium levels and survival prognosis. Second, data related to patient dialysis, such as inter-dialysis weight gain and dialysate concentration, were missing, and it was not possible to exclude the possible effects of disturbances in such factors. Third, it was not possible to determine the exact cause of hyponatremia and hypernatremia and to further verify whether the high mortality rate in patients with hyponatremia and hypernatremia could be reduced by specific interventions or treatments.

## Conclusions

During dialysis in MHD patients, the probability of early hyponatremia was 54.1% versus 6.3% for hypernatremia. Among long-term dialysis, 1.8% of patients had a mean SNa level <135 mmol/L while 0.9% >145 mmol/L. There was a U-shaped relationship between long-term SNa level and the risk of all-cause mortality, with decreased (≤137.5 mmol/L) or elevated (>142.5 mmol/L) SNa levels being an independent risk factor for all-cause mortality in patients with MHD. In long-term dialysis, the greater the fluctuation in a patient’s SNa level, the worse the prognosis.

## Institutional review board statement

The study was conducted according to the guidelines of the declaration of Helsinki, and approved by the Research Ethics Board of the First Affiliated Hospital of Zhejiang University (IIT20230183A).

## Supplementary Material

Supplemental Material
