# Supplementary material for: Effect of long-term serum sodium levels on the prognosis of patients on maintenance hemodialysis
Source: Ren Fail. 2024 Feb 18;46(1):2314629. doi: 10.1080/0886022X.2024.2314629 (PMC10878331; doi:10.1080/0886022X.2024.2314629)
Supplement: Supplemental Material [file IRNF_A_2314629_SM2027.pdf]

Supplementary Table

Table 1 Cox regression analysis of long-term serum sodium levels and all-cause mortality after grouping by glucose (N=15851)

Na (mmol/L)	Glu>7mmol/L (N=11495)	
	HR (95%CI)	P-value
140<Na≤142.5	1	
Na<135	1.485 (1.021-2.159)	0.039
135≤Na≤137.5	1.125 (0.965-1.312)	0.132
137.5<Na≤140	1.034 (0.928-1.152)	0.547
142.5<Na≤145	1.289 (1.101-1.510)	0.002
Na>145	2.467 (1.728-3.523)	<0.001

HR, hazard ratio; CI, confidence interval

Model: Corrected variables included gender, age, primary disease, comorbidities, and vascular access;
